# Single-Step Methodology for Genomic Evaluation in Turkeys (*Meleagris gallopavo*)

**DOI:** 10.3389/fgene.2019.01248

**Published:** 2019-12-20

**Authors:** Emhimad E. A. Abdalla, Flavio S. Schenkel, Hakimeh Emamgholi Begli, Owen W. Willems, Pieter van As, Ryley Vanderhout, Benjamin J. Wood, Christine F. Baes

**Affiliations:** ^1^Centre for Genetic Improvement of Livestock, University of Guelph, Guelph, ON, Canada; ^2^School of Veterinary Science, University of Queensland, Gatton, QLD, Australia; ^3^Hendrix Genetics Research Technology & Service B.V., Boxmeer, Netherlands; ^4^Hybrid Turkeys, Kitchener, ON, Canada; ^5^Institute of Genetics, Vetsuisse Faculty, University of Bern, Bern, Switzerland

**Keywords:** single-step blending, genomic selection, pedigree best linear unbiased prediction, genetic correlation, accuracy, bias

## Abstract

Genomic information can contribute significantly to the increase in accuracy of genetic predictions compared to using pedigree relationships alone. The main objective of this study was to compare the prediction ability of pedigree-based best linear unbiased prediction (PBLUP) and single-step genomic BLUP (ssGBLUP) models. Turkey records of feed conversion ratio, residual feed intake, body weight, breast meat yield, and walking ability were provided by Hybrid Turkeys, Kitchener, Canada. This data was analyzed using pedigree-based and single-step genomic models. The genomic relationship matrix was calculated either using observed allele frequencies, all allele frequencies equal to 0.5 or with a different scaling. To avoid potential problems with inversion, three different weighting factors were applied to combine the genomic and pedigree matrices. Across the studied traits, ssGBLUP had higher heritability estimates and significantly outperformed PBLUP in terms of accuracy. Walking ability was genetically negatively correlated to body weight and breast meat yield; however, it was not correlated to feed conversion ratio (FCR) or residual feed intake (RFI). Body weight showed a moderate positive genetic correlation to feed conversion ratio, residual feed intake and breast meat yield. Feed conversion ratio was strongly correlated to residual feed intake (0.68 ± 0.06). There was almost no genetic correlation between breast meat yield and feed efficiency traits. Larger differences in accuracy between PBLUP and ssGBLUP were observed for traits with lower heritability. Results of the three weighting factors showed only slight differences and an increase in accuracy of prediction compared to PBLUP. Slightly different levels of bias were observed across the models, but were higher among the traits; BMY was the only trait that had a regression coefficient higher than 1 (1.38 to 1.41). We show that incorporating genomic information increases the prediction accuracy for preselection of young candidate turkeys for the five traits investigated. Single-step genomic prediction showed substantially higher accuracy estimates than the pedigree-based model, and only slight differences in bias were observed across the alternate models.

## Introduction

The traditional mixed model equations (Henderson, 1975) are a versatile approach in animal breeding for fitting a variety of models and have been successful for many decades. In such models, genetic effects are estimated based on the covariance structure proportional to the relationship matrix based on pedigree information. This method provides predictions for all animals in the pedigree by adjusting for fixed and random non-genetic effects. Studies have shown that genomic selection, based on information from high-density single nucleotide polymorphism (SNP) markers, accelerates genetic gain by increasing the accuracy of prediction, shortening generation intervals, and improving control of inbreeding (Meuwissen et al., 2001; VanRaden et al., 2009; Verbyla et al., 2009).

Incorporating genomic information in genetic evaluations was initially based on the two-step technique (e.g., VanRaden et al., 2009; Hayes et al., 2009a). This technique includes defining a response variable (e.g., de-regressed estimated breeding values; Garrick et al., 2009) for the genotyped animals, training the markers effects and applying a genomic prediction procedure based on the estimated marker effects for genotyped animals, usually the selection candidates. This analysis is advantageous for providing predictions for genotyped animals, however, obtaining predictions for non-genotyped animals is not straight forward (Aguilar et al., 2010; Christensen and Lund, 2010; Fragomeni et al., 2015). Additional methods have been developed, for example, blending genomic and pedigree information and using other techniques such as correlated traits, selection index and external expected progeny differences; however, each of these methods has its own disadvantages (Garrick and Fernando, 2014). Additional downsides of the two-step approach are that finding a suitable response variable, such as deregressed proofs, as well as an appropriate statistical modeling approach, such as Bayes A, can be challenging (Garrick et al., 2009; Gianola et al., 2009). In addition, the omission of information from non-genotyped animals and selection bias based on availability of genomic information may affect the analyses (Misztal et al., 2009).

Alternatively, a single-step method that combines genomic and pedigree data in a unified analysis has been introduced in the last decade (Legarra et al., 2009; Misztal et al., 2009; Aguilar et al., 2010). This single-step GBLUP closely resembles the traditional genetic evaluation approach based on linear mixed model analyses, and can readily use the available linear algebra machinery and software. In order to be integrated into one single matrix with the numerator relationship matrix (**A**), the genomic relationship matrix (**G**) should be positive definite. Condensing the **G** matrix to remove duplicate rows and columns (e.g. as in Baes and Reinsch, 2007) can be considered to make **G** invertible if the number of markers is higher than the number of individuals. VanRaden, (2008) suggested an improved non-singular matrix (**G** *_w_*) that can be obtained as *w* **G** + (*1* - *w*)**A**_22_, where **A**_22_ is the numerator relationship matrix among genotyped individuals. Such adjustment can be interpreted as the relative weight on the polygenic effect (Christensen and Lund, 2010). In addition to facilitating inversion, values for *w* between 0.90 and 1 showed changes in accuracy of predictions (VanRaden, 2008). Christensen and Lund (2010) reported negligible differences in estimated breeding values when *w* ranged between 0.95 and 0.98.

Genomic selection has been widely adopted in various livestock species (e.g., Hayes et al., 2009b; Wolc et al., 2016; Abdalla et al., 2019). Incorporating genomic information into turkey breeding programs is expected to increase the accuracy of prediction and outperform the pedigree-based method. Genomic-based selection programs reduce the exclusive reliance on the phenotypic, pedigree, and progeny information (Dekkers, 2004; Bolormaa et al., 2013). Moreover, direct selection can be applied to phenotypes that are expensive to measure (e.g., feed efficiency), only expressed late in life (e.g., walking score) and can only be collected once the animal is dead (e.g., breast meat yield). It is also important to determine the possibility of performing simultaneous selection for genetically correlated traits and understanding the response that will occur in each individual trait. The aim of this study was to estimate heritability and genetic correlations for 5 economically important traits measured in turkeys (two feed efficiency traits, body weight, breast meat yield, and walking ability), and to contrast single-step genomic and pedigree-based models in terms of prediction ability using those traits.

## Material and Methods

### Phenotype and Genotype Data

The data used in this study was provided by Hybrid Turkeys, Kitchener, Canada and consisted of 5,619 records on feed conversion ratio (FCR) and residual feed intake (RFI) in males; 170,844 records of body weight (BW) and walking score (WS) at 20 weeks for males (71,012) and 18 weeks for females (99,832); 9,634 records for breast meat yield (BMY) in males as shown in **Table 1**. These records were collected for 10 generations on birds hatched between 2009 and 2017. The birds were reared under a standard feeding system with group housing and shared feeders and drinkers until 15 weeks of age. From 15 weeks of age until 19 to 20 weeks of age, a real-time automated system was used to monitor individual feed intake. This monitoring system consisted of hardware and software subsystems. The bird identification numbers were automatically scanned to detect their identification in each time they entered the feed station and the feeder weight and body weight were recorded using a scale throughout this period (Tu et al., 2011). The FCR was calculated as total feed intake divided by weight gain. To calculate RFI, expected feed intake was obtained as a multiple regression of observed feed intake on metabolic mid-weight, body weight gain and hatch week (Case et al., 2012). At 18 and 20 weeks of age, females and males, respectively, were given a WS between 1 and 6 based on their walking ability, such that animals with a higher walking score had stronger legs and better walking ability. Finally, BMY was taken after slaughtering the males at 21 or 22 weeks and was calculated as:

$$\text{BMY}=\frac{\text{Breast meat weight}}{\text{Live body weight at slaughter}}\times 100.$$

**Table 1:** Descriptive statistics of the analyzed data set, including the number of records, mean and standard deviation of the traits and number of records in the training and validation subsets.

Trait	Number	Mean	Std	Training		Validation	
				Not genotyped	Genotyped	Not genotyped	Genotyped
Feed conversion ratio (kg/kg)	5,592	2.58	0.39	2,711	2,307	464	110
Residual feed intake (kg)	5,592	0	2.51	2,711	2,307	464	110
Body weight (kg)	170,844	17.50	5.32	139,061	13,862	16,783	1,138
Breast meat yield (%)	9,634	24.37	2.33	7,877	843	778	136
Walking score (1 - 6)	170,844	2.10	0.86	139,061	13,862	16,783	1,138

A subset of animals in this population were genotyped using a proprietary 65K single nucleotide polymorphisms (SNP) panel (65,000 SNP; Illumina, Inc.) as shown in **Table 1**. In addition, a pedigree of 783,298 animals was available for analysis. The data was not imputed and as a quality control, SNP markers were excluded if they significantly deviated from Hardy Weinberg proportions (*P*<1×10^-8^), had minor allele frequency lower than 5%, call rate lower than 90%, or were located in non-autosomal regions. After editing, the number of markers remaining for subsequent genomic analysis was 53,455. All animals had a call rate higher than 90%.

### Data Analysis

#### Pedigree-Based Best Linear Unbiased Prediction (PBLUP)

A traditional animal model procedure based on BLUP was used to obtain estimated breeding values for all animals in the pedigree across all traits. The following multi-trait model was used to estimate genetic parameters:

y=Xb+Zu+e,

where **y** is a vector of observations of FCR, RFI, BMY, BW, and WS sorted within animals; **b** is a vector with the fixed effects of hatch week-year and sex for BW and WS and only hatch week-year for FCR, RFI and BMY; **u** is a vector of additive genetic effects (i.e., breeding values), distributed as **u ∼** *N*(**0**, **A**⊗**R**), where **A** is the numerator relationship matrix including the inbreeding coefficients and **R** is the additive genetic variance-covariance matrix among traits; **e** is a vector of residual effects, distributed as $e\sim N(0,\sum_{i}^{+}E_{iy})$ where **E**_iy_ indicates a *m* *_i_*×*m* *_i_* matrix corresponding to the traits that were present for animal *i*, and *m* *_i_* is the number of traits present for animal *i*; **X** and **Z** are incidence matrices for the respective fixed and random effects. Variance components and genetic correlations among the traits were estimated by restricted maximum likelihood (REML) and the procedure was carried out using the BLUPF90 family of programs (Misztal et al., 2014). Inbreeding coefficients were included in REML and BLUP for both pedigree-based and single-step analyses.

#### Single-Step Genomic Best Linear Unbiased Prediction (ssGBLUP)

A combined pedigree and genomic relationship matrix (**H**; Misztal et al., 2009; Aguilar et al., 2010) was created to replace the pedigree-derived relationship matrix (**A**) in the traditional animal model described above and the variance components were estimated based on the **H** matrix, which was created as follows:

$$H^{-1}=A^{-1}+\left[\begin{array}{cc} 0 & 0\\ 0 & {\left(wG+(1-w)A_{22}\right)}^{-1}-A_{22}^{-1} \end{array}\right].$$

In the above, *w* is a constant denoted as a weighting factor in this study as described later, **G** is the genomic relationship matrix, which was calculated as described in VanRaden (2008):

$$G=\frac{(M-P){\left(M-P\right)}^{'}}{2\sum p_{j}(1-p_{j})},$$

where **M** is the matrix of genotypes, with columns representing markers and rows representing individuals. Each element in **M** *_ij_* was coded as 0, 1 or 2 if the genotype of individual *i* for SNP *j* was homozygous for the first allele, heterozygous, or homozygous for the second allele, respectively. **P** is a matrix with average allele frequencies calculated as 2(p_i_ − 0.5), where p_i_ is the frequency of the second allele at locus (column) *i*. If allele frequencies are not properly estimated, biased relationships and consequently biased genomic estimated breeding values (GEBV) may result (Aguilar et al., 2010; Christensen and Lund, 2010). In this sense, allele frequencies need to be estimated from the unselected base population. Since this historical data was not available in the present study, approximations were used. Different allele frequency scenarios were considered. First, allele frequencies were all assumed equal to 0.5 (G0.5F). Second, average allele frequencies calculated from the observed genotype data were used (GObsF). Finally, using observed allele frequencies with different scaling (GSca), following Gianola et al. (2009)

$$\text{GSca = }\frac{\left(M-P\right)\text{ (}M-P{)}^{'}}{\left[{\left({\text{p}}_{0}{\text{- q}}_{0}\right)}^{2}+2\left[\frac{\sum_{j=1}^{\text{m}}{\text{p}}_{\text{j}}({1-p}_{\text{j}})}{\text{m}}\right]\left[\frac{\hat{\alpha }+\hat{\beta }+2}{\hat{\alpha }+\hat{\beta }}\right]\right]\text{m}},$$

where p_0_=α/(α+β) is the expected allele frequency, q_0_=(1-p_0_), α and β are parameters of the beta distribution fitting the allelic frequency, and m is the number of markers. The two unknown parameters α and β were estimated using the method of moments (Johnson et al., 1994) as follows:

$$\hat{\alpha }=\bar{x}\left(\frac{\bar{x}\left(1-\bar{x}\right)}{\bar{v}}-1\right).$$

$$\hat{\beta }=\left(1-\bar{x}\right)\left(\frac{\bar{x}\left(1-\bar{x}\right)}{\bar{v}}-1\right),$$

In the above, $\bar{x}$ and $\bar{v}$ are the mean and the variance of allele frequencies, respectively. To avoid potential problems with inversion and to account for the relative weight of the polygenic effect needed to explain the total additive variance, three different values (0.95, 0.90 and 0.85) have been considered for *w* in this study. These three alternative values for *w* are denoted as ssGBLUP_0.95, ssGBLUP_0.90 and ssGBLUP_0.85, respectively.

### Assessment of Accuracy

The following technique was used to compare prediction ability of the PBLUP and the ssGBLUP models. First, adjusted phenotypes corrected for fixed effects were obtained (adjusted phenotypes) for all animals based on the PBLUP model (PBLUP full model). Second, approximately 10% of the youngest, phenotyped birds had their records set to missing (PBLUP reduced model). The estimated breeding values (EBV) for this 10% of animals were then estimated based on their parent average and comprised the validation group as shown in **Table 1**. Next, the accuracy of the PBLUP model was calculated as the Pearson correlation coefficient between the adjusted phenotypes of the animals in the validation group from the PBLUP full model and their corresponding EBV obtained from the PBLUP reduced model. The same procedure and validation data set used to validate the PBLUP was used for ssGBLUP, but the accuracy was calculated as the Pearson correlation coefficient between the adjusted phenotypes of the animals in the validation group from the PBLUP full model and their corresponding (G)EBV obtained from the ssGBLUP reduced model.

### Assessment of Bias

For PBLUP, the bias of predictions and their associated standard errors were assessed by the regression of each adjusted phenotype from the full PBLUP model in the validation subset on the corresponding EBV from the reduced PBLUP model. For ssGBLUP, the bias was evaluated as the regression of the adjusted phenotype from the full PBLUP model on the corresponding (G)EBV obtained from the reduced ssGBLUP model. Slope coefficients from the linear regression with values closer to one are preferred, as slope coefficients notably less than or higher than 1 reflect inflation and deflation, respectively (Su et al., 2012; González-Recio et al., 2014).

## Results and Discussion

### Allele Frequencies to Estimate G

The three different allele frequency approximations (i.e., G0.5F, GObsF, and GSca) showed similar results in terms of accuracy and bias and hence the observed allele frequencies (GObsF) were considered for subsequent analyses.

### Genetic Parameters

#### Heritabilities

Estimates of heritability from multi-trait PBLUP and alternatively weighted ssGBLUP are shown in **Table 2**. Based on pedigree, the heritability estimate for body weight was 0.35 ± 0.06. Similar results of body weight heritability in turkey have been reported (e.g., Nestor et al., 1999; Aslam et al., 2011). The heritability for BMY was 0.27 ± 0.04 and slightly lower than the estimates reported by Aslam et al. (2011) and Le Bihan-Duval et al. (2008) in turkeys (0.30 ± 0.10) and chicken (0.30 ± 0.04), respectively. The heritability estimate for FCR (0.14 ± 0.02) was similar to those reported by Case et al. (2012) (0.16 ± 0.02) and for RFI (0.12 ± 0.02) was lower than the value reported by Case et al. (2012) (0.21 ± 0.02). Across all traits, ssGBLUP had higher heritability estimates than PBLUP. The heritabilities for FCR, RFI, BW, BM and WS were 0.17 ± 0.03, 0.15 ± 0.02, 0.41 ± 0.06, 0.30 ± 0.05, and 0.26 ± 0.04 for ssGBLUP_0.90 model, respectively, while the estimates were 0.14 ± 0.02, 0.14 ± 0.02, 0.35 ± 0.06, 0.27 ± 0.04, and 0.24 ± 0.04 for the PBLUP model.

**Table 2:** Heritability estimates ± standard errors for the studied traits based on the pedigree-based best linear unbiased prediction (PBLUP) and the single-step genomic best linear unbiased prediction blending (ssGBLUP) with three different combinations of blending weights (*w*) for genomic (**G**) and pedigree (**A**_22_) relationship matrices.

Model^1^	PBLUP	ssGBLUP_0.95	ssGBLUP_0.90	ssGBLUP_0.85
Feed conversion ratio	0.14 ± 0.02	0.17 ± 0.03	0.17 ± 0.03	0.17 ± 0.03
Residual feed intake	0.12 ± 0.02	0.15 ± 0.02	0.15 ± 0.02	0.15 ± 0.02
Body weight	0.35 ± 0.06	0.41 ± 0.06	0.41 ± 0.06	0.40 ± 0.06
Breast meat yield	0.27 ± 0.04	0.30 ± 0.05	0.30 ± 0.05	0.30 ± 0.05
Walking score	0.24 ± 0.04	0.26 ± 0.04	0.26 ± 0.04	0.26 ± 0.05

^1^ssGBLUP_0.95: w = 0.95; ssGBLUP_0.90: w = 0.90; ssGBLUP_0.85: w = 0.85.

#### Genetic and Residual Correlations

Accurate estimation of genetic correlations among traits is imperative for any breeding program (Pantelić et al., 2011). Genetic and residual correlations given by the overall most accurate method (ssGBLUP_0.90, see next sections) are shown in **Table 3**. The genetic correlations between BW and FCR (0.19 ± 0.07), RFI (0.13 ± 0.08) and BMY (0.16 ± 0.06) were all positive and weak, but BW was moderately negatively correlated to WS (-0.47 ± 0.02). Low or no genetic correlation was observed between BMY and FCR (-0.05 ± 0.01); WS and FCR (-0.09 ± 0.07); RFI and BMY (0.09 ± 0.01). Walking ability was also moderately negatively correlated to BMY (-0.35 ± 0.05) and lowly correlated to RFI (0.08 ± 0.01). The two feed efficiency traits were highly positively correlated (0.68 ± 0.06). The residual correlation of BW was negative with FCR (-0.39 ± 0.01) and with RFI (-0.17 ± 0.03), but positive with BMY (0.19 ± 0.02). Walking ability, on the other hand, had negative correlation (-0.15 ± 0.01) with BW and approximately zero residual correlations with all other studied traits. BMY showed low negative residual correlation with FCR (-0.10 ± 0.01) and RFI (-0.07 ± 0.03), and a positive moderate (0.23 ± 0.09) residual correlation was found between FCR and RFI.

**Table 3:** Genetic (above diagonal) and residual (below diagonal) correlations ± standard errors for the studied traits based on the single-step genomic best linear unbiased prediction (ssGBLUP_0.90).

	Feed conversion ratio	Residual feed intake	Body weight	Breast meat yield	Walking score
Feed conversion ratio		0.68 ± 0.06	0.19 ± 0.07	-0.05 ± 0.01	-0.09 ± 0.07
Residual feed intake	0.23 ± 0.09		0.13 ± 0.08	-0.13 ± 0.01	0.08 ± 0.01
Body weight	-0.39 ± 0.01	-0.17 ± 0.03		0.16 ± 0.06	-0.35 ± 0.05
Breast meat yield	-0.10 ± 0.01	-0.07 ± 0.03	0.19 ± 0.02		-0.47 ± 0.02
Walking score	0.03 ± 0.00	0.05 ± 0.01	-0.15 ± 0.01	-0.06 ± 0.01	

These results indicate a moderate and unfavorable genetic correlation between WS with both BW and BMY, meaning that selection for increased BW and BMY may also increase leg problems. The genetic increase in body weight of turkeys starting at age 16 weeks was previously found associated with frequency of leg abnormalities (Nestor, 1984). The main cause for this issue is that the direct selection for increased BMY and the overall BW had led to a relatively faster increase in these parts of the body than the muscles and bones of the legs (Nestor et al., 1987). Since the estimated unfavorable genetic correlations are only moderate, it would be desirable to further investigate these traits to detect quantitative trait loci/genes that may enhance both BW/BMY and walking ability. This could be performed in the context of genome-wide association analysis with multiple traits using, for example, structural equation modeling approaches (Rosa et al., 2011). Conversely, BW was positively correlated to feed efficiency traits as well as to BMY, indicating that selection for higher BW could indirectly increase these two traits. The genetic correlations between feed efficiency traits and walking ability were close to zero. No previous studies were available to compare these results. The high genetic correlation between FCR and RFI is expected and agrees with previous estimates in the turkey (Case et al., 2012), beef (Schenkel et al., 2004) and pigs (Hoque et al., 2007).

### Accuracy of Prediction

Across the studied traits, the ssGBLUP model outperformed PBLUP (shown in **Table 4**). Accuracies of PBLUP ranged from 0.21 to 0.36 and those for ssGBLUP ranged from 0.26 to 0.40 with lower heritability traits showing a higher increase in accuracy between PBLUP and ssGBLUP. Results of the ssGBLUP_90 showed an increase in accuracy of 31%, 29%, 12%, 23%, and 16% for FCR, RFI, BW, BMY, and WS, respectively. In general, marker-based EBV have shown higher predictive ability than those estimates using pedigree relationships (e.g., Daetwyler et al., 2007; Hayes et al., 2010). The results of this study indicate that incorporating genomic data (ssGBLUP blending) in the prediction of the traits investigated here produces more accurate predictions, which will lead to an improved genetic gain in turkey breeding.

**Table 4:** Accuracy (Pearson correlation coefficient) of estimated breeding values for the studied traits based on the pedigree-based best linear unbiased prediction (PBLUP) and the single-step genomic best linear unbiased prediction prediction (ssGBLUP) with three different combinations of blending weights (*w*) for genomic (**G**) and pedigree (**A**_22_) relationship matrices.

Model^1^	PBLUP	ssGBLUP_0.95	ssGBLUP_0.90	ssGBLUP_0.85
Feed conversion ratio	0.29	0.38	0.38	0.37
Residual feed intake	0.21	0.26	0.27	0.26
Body weight	0.36	0.40	0.40	0.39
Breast meat yield	0.30	0.37	0.37	0.36
Walking score	0.26	0.30	0.30	0.30

^1^ssGBLUP_0.95: w = 0.95; ssGBLUP_0.90: w = 0.90; ssGBLUP_0.85: w = 0.85.

In practice, there could be a number of issues with single-step prediction. The genomic matrix can be singular if there are more genotyped animals than markers, or in the presence of clones (Misztal et al., 2009; Aguilar et al., 2010; Forni et al., 2011). More generally, collinearity between variables and low rank (row or column, equivalently) of the matrix are reasons that make the inversion of a matrix difficult or not possible. To avoid the potential problems with inversion and to account for the relative weight of the polygenic effect needed to explain the total additive variance, different scenarios were applied to scale the genomic matrix to the pedigree matrix. As shown in **Table 4**, the three weights applied to the ssGBLUP model in the present study showed comparable prediction abilities. Using simulated data, Vitezica et al. (2010) reported that the single-step method showed less bias and more accurate prediction when the genomic relationship matrix was adjusted by a constant. Similar results were reported by Chen et al. (2011) and by Forni et al. (2011) using chicken and pig data, respectively. Although the differences between the weightings applied in this study were not large, the weighting factors of 0.95 and 0.90 could be the optimal choices to improve breast meat yield and the other four traits, respectively.

### Assessment of Prediction Bias

In addition to the accuracy of prediction, it is important to examine potential bias when establishing a breeding program or comparing models. Although it does not change the ranking of animals from the same generation, systematic over- or under-estimation of genetic merit leads to consistent biases (González-Recio et al., 2014). Hence, the predicted performance of the selection candidates could be over- or under-estimated. The regression coefficients for all traits across the different models considered in this study are shown in **Table 5**. A regression coefficient less or greater than one indicates an overestimation (inflation) or underestimation (deflation), respectively. In general, bias estimates were quite similar among models, but varied among trait, and the bias estimates ranged between 0.73 ± 0.04 and 1.41 ± 0.21. Except for BMY, all traits had regression coefficients lower than one. Among the models, PBLUP showed the highest deflation for breast meat yield (1.41 ± 0.21), which could be due to the small number of animals with both genotypes and phenotypes for this trait. PBLUP also showed the lowest deflation for feed conversion ratio (0.94 ± 0.17) and for WS (0.73 ± 0.04), however compared to ssBLUP_95 (ssGBLUP_0.95; 0.95 ± 0.17 and 0.75 ± 0.17, respectively), the differences were negligible. The differences among the three ssGBLUP models were also small with some slight changes in bias for individual traits. The BMY had a regression coefficient higher than one and the three ssGBLUP models showed similar but slightly lower inflation (1.38 ± 0.21). Despite the accuracy of any prediction procedure, it is worthwhile to pay attention to bias and apply appropriate bias correction methods, such as including a genomic pseudo-performance based on GEBV for all the selection candidates (Patry and Ducrocq, 2011). This is important if proven and young candidates are expected to be simultaneously selected. The dataset used in this study, particularly the genotyped population for BMY, is small. Hence, with a larger training population, prediction accuracy could be enhanced and bias could be reduced.

**Table 5:** Regression coefficients of estimated breeding values from the full PBLUP model on their corresponding EBV from the reduced PBLUP model and on their corresponding GEBV from the reduced ssGBLUP model with three different combinations of blending weights (*w*) for genomic (**G**) and pedigree (**A**_22_) relationship matrices.

Model^1^	PBLUP	ssGBLUP_0.95	ssGBLUP_0.90	ssGBLUP_0.85
Feed conversion ratio	0.94 ± 0.17	0.95 ± 0.17	0.95 ± 0.17	0.95 ± 0.17
Residual feed intake	0.79 ± 0.12	0.79 ± 0.12	0.80 ± 0.12	0.80 ± 0.12
Body weight	0.82 ± 0.03	0.82 ± 0.03	0.83 ± 0.03	0.82 ± 0.03
Breast meat yield	1.41 ± 0.21	1.38 ± 0.21	1.38 ± 0.21	1.38 ± 0.21
Walking score	0.73 ± 0.04	0.75 ± 0.04	0.75 ± 0.04	0.76 ± 0.04

^1^ssGBLUP_0.95: w = 0.95; ssGBLUP_0.90: w = 0.90; ssGBLUP_0.85: w = 0.85.

## Conclusions

In this study, we estimated heritability, genetic correlations and contrasted single-step genomic and pedigree-based models in terms of prediction ability using two feed efficiency traits, body weight, breast meat yield, and walking ability in turkeys. We showed that incorporating genomic information into breeding programs increased prediction accuracy for the five traits investigated. Single-step genomic prediction showed substantially higher accuracy estimates than the pedigree-based model. Slightly different levels of bias were observed across the alternate models, but a high variation in bias was observed across the traits. The five traits investigated are expensive to measure, therefore the use of genomic selection has strong potential to reduce the cost of turkey breeding programs, as the reliance on phenotypic, pedigree, and progeny information is reduced.

## Data Availability Statement

Data that support the findings of this study are available from Hybrid Turkeys upon reasonable request, but restrictions apply to the availability of these data, which were used under a license of a material transfer agreement for the current study, and thus are not publicly available.

## Ethics Statement

This study was carried out in accordance with the principles of the Canadian Council on Animal Care, the Hendrix Genetics Animal Welfare Policy, and the University of Guelph Animal Care Committee. The protocol was approved by the University of Guelph Animal Care Committee (Animal Use Protocol #3782).

## Author Contributions

The author(s) have made the following declarations about their contributions: Conceived and designed the experiments: EA, FS, CB. Analyzed the data: EA, FS. Interpreted results: EA, CB, HE, FS, BW, OW, PV. Wrote the paper: EA, CB, FS, RV, BW.

## Funding

This study was conducted as part of the project entitled ‘Application of genomic selection in turkeys for health, welfare, efficiency and production traits’. This Project was funded by the Government of Canada through Genome Canada and the Ontario Genomics Institute (OGI-133) through the Genome Canada Genomic Application Partnership Program [recipients: CB (Academic), BW (Industry)]. This study was also financially supported by Hybrid Turkeys (Kitchener, Canada). The authors declare that this study received funding from Hybrid Turkeys. The funder was involved in providing datasets used in this study.

## Conflict of Interest

BW and OW are employed by Hybrid Turkeys, Kitchener Canada. PV is employed by Hendrix Genetics, Boxmeer, Netherlands.

The remaining authors declare that the research was conducted in the absence of any commercial or financial relationships that could be construed as a potential conflict of interest.

## References

[B1] Abdalla E. A. Lopes F. B. Byrem T. M. Weigel K. A. Rosa G. J. M. (2019). Genomic prediction of bovine leukosis incidence in a US Holstein population. Livest. Sci. 225, 73–77. 10.1016/j.livsci.2019.05.004

[B2] Aguilar I. Misztal I. Johnson D. L. Legarra A. Tsuruta S. Lawlor T. J. (2010). Hot topic: a unified approach to utilize phenotypic, full pedigree, and genomic information for genetic evaluation of Holstein final score1. J. Dairy Sci. 93, 743–752. 10.3168/jds.2009-2730 20105546

[B3] Aslam M. L. Bastiaansen J. W. M. Crooijmans R. P. M. A. Ducro B. J. Vereijken A. Groenen M. A. M. (2011). Genetic variances, heritabilities and maternal effects on body weight, breast meat yield, meat quality traits and the shape of the growth curve in turkey birds. BMC Genet. 12, 14. 10.1186/1471-2156-12-14 21266032 PMC3039623

[B4] Baes C. Reinsch N. (2007). Computing the condensed conditional gametic QTL relationship matrix and its inverse. Arch. Anim. Breed. 50, 294–308. 10.5194/aab-50-294-2007

[B5] Bolormaa S. Pryce J. E. Kemper K. Savin K. Hayes B. J. Barendse W. (2013) Accuracy of prediction of genomic breeding values for residual feed intake and carcass and meat quality traits in Bos taurus, Bos indicus, and composite beef cattle1. J. Anim. Sci. 91, 3088–3104. 10.2527/jas.2012-5827 23658330

[B6] Case L. A. Wood B. J. Miller S. P. (2012). The genetic parameters of feed efficiency and its component traits in the turkey (*Meleagris gallopavo*). Genet. Sel. Evol. 44, 2. 10.1186/1297-9686-44-2 22268922 PMC3296663

[B7] Chen C. Y. Misztal I. Aguilar I. Legarra A. Muir W. M. (2011). Effect of different genomic relationship matrices on accuracy and scale. J. Anim. Sci. 89, 2673–2679. 10.2527/jas.2010-3555 21454868

[B8] Christensen O. F. Lund M. S. (2010). Genomic prediction when some animals are not genotyped. Genet. Sel. Evol. 42, 1–8. 10.1186/1297-9686-42-2 20105297 PMC2834608

[B9] Daetwyler H. D. Villanueva B. Bijma P. Woolliams J. A. (2007). Inbreeding in genome-wide selection. J. Anim. Breed. Genet. 124, 369–376. 10.1111/j.1439-0388.2007.00693.x 18076474

[B10] Dekkers J. C. M. (2004). Commercial application of marker-and gene-assisted selection in livestock: strategies and lessons. J. Anim. Sci. 82, E313–E328. 10.2527/2004.8213_supplE313x 15471812

[B11] Forni S. Aguilar I. Misztal I. (2011). Different genomic relationship matrices for single-step analysis using phenotypic, pedigree and genomic information. Genet. Sel. Evol. 43, 1–7. 10.1186/1297-9686-43-1 PMC3022661 21208445

[B12] Fragomeni B. O. Lourenco D. A. L. Tsuruta S. Masuda Y. Aguilar I. Legarra A. (2015). Hot topic: Use of genomic recursions in single-step genomic best linear unbiased predictor (BLUP) with a large number of genotypes. J. Dairy Sci. 98, 4090–4094. 10.3168/jds.2014-9125 25864050

[B13] Garrick D. J. Fernando R. L. (2014). Genomic prediction and genome-wide association studies in beef and dairy cattle. 2nd ed Eds. D. J. Garrick, and A. Ruvinsky (Wallingford, UK: CABI Publishing).

[B14] Garrick D. J. Taylor J. F. Fernando R. L. (2009). Deregressing estimated breeding values and weighting information for genomic regression analyses. Genet. Sel. Evol. 41, 1–8. 10.1186/1297-9686-41-55 20043827 PMC2817680

[B15] Gianola D. de los Campos G. Hill W. G. Manfredi E. Fernando R. (2009). Additive genetic variability and the Bayesian alphabet. Genetics 183, 347–363. 10.1534/genetics.109.103952 19620397 PMC2746159

[B16] González-Recio O. Rosa G. J. M. Gianola D. (2014). Machine learning methods and predictive ability metrics for genome-wide prediction of complex traits. Livest. Sci. 166, 217–231. 10.1016/j.livsci.2014.05.036

[B17] Hayes B. J. Bowman P. J. Chamberlain A. C. Verbyla K. Goddard M. E. (2009a). Accuracy of genomic breeding values in multi-breed dairy cattle populations. Genet. Sel. Evol. 41, 1–9. 10.1186/1297-9686-41-1 19930712 PMC2791750

[B18] Hayes B. J. Chamberlain A. J. Bowman P. J. Goddard M. E. (2009b). Invited review: genomic selection in dairy cattle: progress and challenges. J. Dairy Sci. 92, 433–443. 10.3168/jds.2008-1646 19164653

[B19] Hayes B. J. Pryce J. Chamberlain A. J. Bowman P. J. Goddard M. E. (2010). Genetic architecture of complex traits and accuracy of genomic prediction: coat colour, milk-fat percentage, and type in Holstein cattle as contrasting model traits. PloS Genet. 6, e1001139. 10.1371/journal.pgen.1001139 20927186 PMC2944788

[B20] Henderson C. R. (1975). Best linear unbiased estimation and prediction under a selection model. Biometrics 31, 423–447. 10.2307/2529430 1174616

[B21] Hoque M. A. Kadowaki H. Shibata T. Oikawa T. Suzuki K. (2007). Genetic parameters for measures of the efficiency of gain of boars and the genetic relationships with its component traits in Duroc pigs1. J. Anim. Sci. 85, 1873–1879. 10.2527/jas.2006-730 17431052

[B22] Johnson N. L. Kotz S. Balakrishnan N. (1994). “Chapter 21: Beta Distributions,” in Continuous univariate distributions, 2nd ed (New York U. S. A.: Wiley).

[B23] Le Bihan-Duval E. Debut M. Berri C. M. Sellier N. Santé-Lhoutellier V. Jégo Y. (2008). Chicken meat quality: genetic variability and relationship with growth and muscle characteristics. BMC Genet. 9, 1–6. 10.1186/1471-2156-9-53 18706119 PMC2533670

[B24] Legarra A. Aguilar I. Misztal I. (2009). A relationship matrix including full pedigree and genomic information. J. Dairy Sci. 92, 4656–4663. 10.3168/jds.2009-2061 19700729

[B25] Meuwissen T. H. Hayes B. J. Goddard M. E. (2001). Prediction of total genetic value using genome-wide dense marker maps. Genetics 157, 1819–1829. 11290733 10.1093/genetics/157.4.1819 PMC1461589

[B26] Misztal I. Legarra A. Aguilar I. (2009). Computing procedures for genetic evaluation including phenotypic, full pedigree, and genomic information. J. Dairy Sci. 92, 4648–4655. 19700728 10.3168/jds.2009-2064

[B27] Misztal I. Tsuruta S. Lourenco D. Aguilar I. Legarra A. Vitezica Z. (2014). Manual for BLUPF90 family of programs. Athens Univ. Georg. U. S. A. 125.

[B28] Nestor K. E. Bacon W. L. Moorhead P. D. Saif Y. M. Havenstein G. B. Renner P. A. (1987). Comparison of bone and muscle growth in Turkey lines selected for increased body weight and increased shank width. Poult. Sci. 66, 1421–1428. 10.3382/ps.0661421 3684865

[B29] Nestor K. E. Anderson J. W. Patterson R. A. (1999). Genetics of growth and reproduction in the Turkey. 14. Changes in genetic parameters over thirty generations of selection for increased body weight. Poult. Sci. 56, 337–347. 10.3382/ps.0560337 10780636

[B30] Nestor K. E. (1984). Genetics of growth and reproduction in the Turkey.: 9. Long-term selection for increased 16-week body weight. Poult. Sci. 63, 2114–2122. 10.3382/ps.0632114 6514659

[B31] Pantelić V. Sretenović L. Ostojić-Andrić D. Trivunović S. Petrović M. M. Aleksić S. (2011) Heritability and genetic correlation of production and reproduction traits of Simmental cows. Afr. J. Biotechnol. 10, 7117–7121.

[B32] Patry C. Ducrocq V. (2011). Accounting for genomic pre-selection in national BLUP evaluations in dairy cattle. Genet. Sel. Evol. 43, 30. 10.1186/1297-9686-43-30 21851619 PMC3200986

[B33] Rosa G. J. M. Valente B. D. de los Campos G. Wu X.-L. Gianola D. Silva M. A. (2011). Inferring causal phenotype networks using structural equation models. Genet. Sel. Evol. 43, 6. 10.1186/1297-9686-43-6 21310061 PMC3056759

[B34] Schenkel F. S. Miller S. P. Wilton J. W. (2004). Genetic parameters and breed differences for feed efficiency, growth, and body composition traits of young beef bulls. Can. J. Anim. Sci. 84, 177–185. 10.4141/A03-085

[B35] Su G. Madsen P. Nielsen U. S. Mäntysaari E. A. Aamand G. P. Christensen O. F. (2012). Genomic prediction for Nordic Red Cattle using one-step and selection index blending. J. Dairy Sci. 95, 909–917. 10.3168/jds.2011-4804 22281355

[B36] Tu X. Du S. Tang L. Xin H. Wood B. (2011). A real-time automated system for monitoring individual feed intake and body weight of group housed turkeys. Comput. Electron. Agric. 75, 313–320. 10.1016/j.compag.2010.12.007

[B37] VanRaden P. M. Van Tassell C. P. Wiggans G. R. Sonstegard T. S. Schnabel R. D. Taylor J. F. (2009). Invited review: reliability of genomic predictions for North American Holstein bulls. J. Dairy Sci. 92, 16–24. 10.3168/jds.2008-1514 19109259

[B38] VanRaden P. M. (2008). Efficient methods to compute genomic predictions. J. Dairy Sci. 91, 4414–4423. 10.3168/jds.2007-0980 18946147

[B39] Verbyla K. L. Hayes B. J. Bowman P. J. Goddard M. E. (2009). Accuracy of genomic selection using stochastic search variable selection in Australian Holstein Friesian dairy cattle. Genet. Res. (Camb.) 91, 307. 10.1017/S0016672309990243 19922694

[B40] Vitezica Z. G. Aguilar I. Legarra A. (2010). “One-step vs. multi-step methods for genomic prediction in presence of selection,” in Proceedings of the world congress on genetics applied to livestock production, volume genetic improvement programmes: selection using molecular information - lecture sessions, 1–6.

[B41] Wolc A. Kranis A. Arango J. Settar P. Fulton J. E. O’Sullivan N. P. (2016). Implementation of genomic selection in the poultry industry. Anim. Front. 6, 23–31. 10.2527/af.2016-0004

